# Diagnosis and therapy of recurrent renal metastases after surgery for testicular seminoma: a rare case report and review of the literature

**DOI:** 10.3389/fonc.2025.1542368

**Published:** 2025-05-22

**Authors:** Zhuang Li, Yuanyuan Luo, Bo Yu, Hao Su, Dongbo Yuan, Jianguo Zhu

**Affiliations:** ^1^ Department of Urology, Guizhou Provincial People’s Hospital, Guiyang, Guizhou, China; ^2^ Department of Urology, Affiliated Hospital of Guizhou Medical University, Guiyang, Guizhou, China; ^3^ Department of Medical College, Guizhou University, Guiyang, Guizhou, China; ^4^ Department of Urology, the Second Affiliated Hospital of Zunyi Medical University, Zunyi, Guizhou, China

**Keywords:** seminoma, renal metastatic tumor, cryptorchidism, chemotherapy, nephrectomy

## Abstract

**Background:**

Seminoma is a malignant tumor arising from testicular germ cells, with cryptorchidism recognized as a major risk factor for its development. Although retroperitoneal lymph node metastasis is common, renal metastasis is exceedingly rare. Testicular seminomas are generally highly sensitive to chemotherapy; however, the treatment and prognosis become significantly more challenging in cases of distant metastasis.

**Case presentation:**

This case report describes a 39-year-old male patient who unexpectedly discovered a left testicular seminoma following surgery but did not receive postoperative adjuvant chemotherapy due to personal reasons. Thirteen months after surgery, the patient was admitted for left-sided lumbar pain for 1 month. Abdominal enhanced CT imaging revealed a large renal tumor measuring 115 × 120 × 97 mm at the lower pole of the left kidney. The patient subsequently underwent a percutaneous biopsy of the left renal mass, and histopathological examination confirmed that the renal tumor originated from testicular seminoma. The patient then received four cycles of bleomycin, etoposide, and cisplatin (BEP) chemotherapy. Follow-up abdominal CT showed a reduction in the size of the left renal tumor to 56 × 45 × 60 mm, with several enlarged lymph nodes in the abdominal cavity and retroperitoneum (the largest approximately 10 mm in diameter). The patient eventually underwent “left nephrectomy and partial retroperitoneal lymph node dissection”. Postoperative histopathological analysis revealed no evidence of residual tumor cells. Shortly after surgery, the patient developed acute complete intestinal obstruction, which was treated surgically, and his condition improved significantly. The patient maintained clinical stability throughout the 4-month follow-up period without evidence of disease recurrence.

**Conclusion:**

Postoperative adjuvant chemotherapy is essential for patients with testicular seminoma to minimize the risk of tumor recurrence and metastasis. In cases of renal metastasis from seminoma, salvage chemotherapy using the BEP regimen combined with nephrectomy may contribute to improved clinical outcomes.

## 1 Introduction

Testicular tumors account for approximately 1% to 2% of all male malignancies and are the most common solid tumors among male individuals aged 15 to 40 years (1, 2). Germ cell tumors account for 90% to 95% of all testicular cancers, with the major histological subtypes being seminomas and non-seminomas. Established risk factors include cryptorchidism, a positive family history of testicular cancer, gonadal dysgenesis, and Klinefelter syndrome (2). Some studies suggest prolonged heat exposure in cryptorchid testes may induce abnormal apoptosis, allowing a subset of primordial germ cells to evade immune surveillance. Through the accumulation of genetic mutations or cellular imbalance, these cells may develop into germ cell neoplasia *in situ*, ultimately progressing to testicular tumors in adulthood (3, 4). Seminoma is the most common type of tumor in patients with cryptorchidism, accounting for approximately 30% to 40% of all testicular germ cell tumors (5, 6). Previous studies have demonstrated that the pathogenesis of testicular seminoma involves multiple factors, including genetic mutations, gonadal dysgenesis, hormonal imbalances, increased testicular temperature, defects in DNA repair mechanisms, and environmental influences (7, 8).

The treatment of testicular seminoma primarily consists of surgical resection and chemotherapy. While 90% of patients have a favorable prognosis, the tumor has a high propensity for lymphatic metastasis (9). Approximately 80% of seminomas are localized to the testis at diagnosis, with an estimated recurrence rate of 13% to 20% if adjuvant therapy is not administered (10). According to the European Society for Medical Oncology - European Reference Network for Rare Adult Solid Cancers (ESMO-EURACAN) guidelines, active surveillance or combination therapy with bleomycin, etoposide, and cisplatin (BEP regimen) is recommended to prevent postoperative tumor recurrence or metastasis in testicular seminoma (11). Nevertheless, adherence to postoperative chemotherapy varies among patients, which may be related to several factors, such as chemotherapy-related toxicities, financial burden, and lack of patient awareness of the risk of tumor recurrence (12). In addition, the different histological subtypes of seminoma may also impact disease complications and prognosis. Compared to localized seminoma, patients with lymphatic or distant metastasis are more prone to postoperative complications such as thrombosis, immunosuppression, and secondary malignancies (13). This case report describes a patient who underwent a left orchiectomy for cryptorchidism, with postoperative pathology confirming a diagnosis of testicular seminoma. However, the patient did not receive the prescribed standard chemotherapy regimen after surgery due to personal reasons. The patient developed metastases in the abdomen, retroperitoneal lymph nodes, and left kidney within 13 months postoperatively. The patient received four cycles of BEP chemotherapy, followed by a left nephrectomy and partial excision of retroperitoneal enlarged lymph nodes. Fortunately, at the 4-month postoperative follow-up, the patient demonstrated good recovery without signs of recurrence. The patient's full clinical course is outlined in **Table 1**.

**Table 1:** Timeline of clinical management and tumor progression characteristics.

Admission date	Major events	Tumor progression characteristics	Tumor marker levels	Treatment regimen	Histopathological characteristics and therapeutic outcomes
January 2023	A left groin mass persisting for over 1 year, with 1 day of pain	The patient reported no significant change in the size of the left groin mass over the past year	Not tested	Left inguinal incarcerated direct hernia repair, orchiectomy	Testicular seminoma, immunohistochemical profile: CD117 (9.7% positive), Ki-67 (70% proliferation index)
February 2024	Left flank pain persisting for over 1 month	CT revealed a 115 × 120 × 97 mm mass at the lower pole of the left kidney, with multiple enlarged lymph nodes in the abdomen and retroperitoneum	Not tested	Percutaneous core needle biopsy of the left renal mass	Left renal seminoma, immunohistochemistry: PLAP (diffuse +), SALL4 (nuclear +), Ki-67 proliferation index ~60%
March 2024 to July 2024	Metastatic seminoma was diagnosed 10 days ago, with BEP chemotherapy planned	CT surveillance during chemotherapy showed no evidence of new or recurrent tumors	AFP = 6.0 ng/mL, hCG-β < 1.2 U/L, LDH = 177 U/L	The patient completed four cycles of BEP chemotherapy	After completing four cycles of BEP chemotherapy, the tumor volume decreased but was not completely eradicated
October 2024	Post-chemotherapy re-evaluation indicates planned surgical intervention	CT demonstrates a 56 × 45 × 60 mm mass at the lower pole of the left kidney, with regression of abdominal and retroperitoneal lymphadenopathy compared to pre-chemotherapy imaging	AFP = 6.3 ng/mL, hCG-β < 1. 2 U/L, LDH = 176 U/L	Left nephrectomy and partial excision of retroperitoneal enlarged lymph nodes	Postoperative pathological examination revealed no residual tumor cells, indicating a significant chemotherapeutic response
November 2024	Abdominal distension and cessation of flatus/defecation occurred 12 days post left nephrectomy	Abdominal CT showed small bowel gas and fluid accumulation with intestinal dilation, suggesting acute complete intestinal obstruction caused by residual enlarged abdominal lymph nodes and postoperative adhesions following nephrectomy	Not tested	Exploratory laparotomy, resection, and anastomosis of the obstructed small intestine	The patient was discharged after return of normal flatus and bowel movements. Postoperative pathology confirmed no residual tumor cells
March 2025	The patient was readmitted for scheduled postoperative surveillance	Abdominal CT demonstrates no evidence of pathologically enlarged lymph nodes in the peritoneal cavity or retroperitoneal space	AFP = 6.2 ng/mL, hCG-β < 1.2 U/L, LDH = 138 U/L	No specific therapeutic intervention was required at this time	The patient has achieved an excellent recovery with no evidence of tumor recurrence in current evaluation

BEP, bleomycin, etoposide, and cisplatin; AFP, alpha-fetoprotein; hCG-β, human chorionic gonadotropin; LDH, lactate dehydrogenase.

## 2 Case description

The patient was a 39-year-old unmarried and childless man with a low educational level. He presented to a local medical institution on 16 January 2023 with a chief complaint of a left inguinal mass that had persisted for over 1 year. During this period, the mass remained stable in size and did not cause significant discomfort.

On the day preceding admission, the patient experienced a sudden onset of severe, persistent pain in the left inguinal region, associated with localized swelling and tenderness. The patient reported no previous history of similar pain episodes, traumatic injuries, or identifiable predisposing factors. The patient was diagnosed with an incarcerated left inguinal hernia and left cryptorchidism (previously undiagnosed) based on clinical evaluation at the local hospital. Surgical management included left incarcerated direct hernia repair combined with orchiectomy, with subsequent histopathological examination of the resected specimen. Postoperative pathology confirmed testicular seminoma, with immunohistochemical staining demonstrating the following profile: alpha-fetoprotein (AFP) (−), CD117 (focal positivity, 9.7%), CK-P (−), Ki-67 (70% positive), and Inhibin-α (−). Although adjuvant chemotherapy was recommended as part of the standard treatment protocol, the patient initially declined further therapeutic intervention at that time.

**Figure 1:**
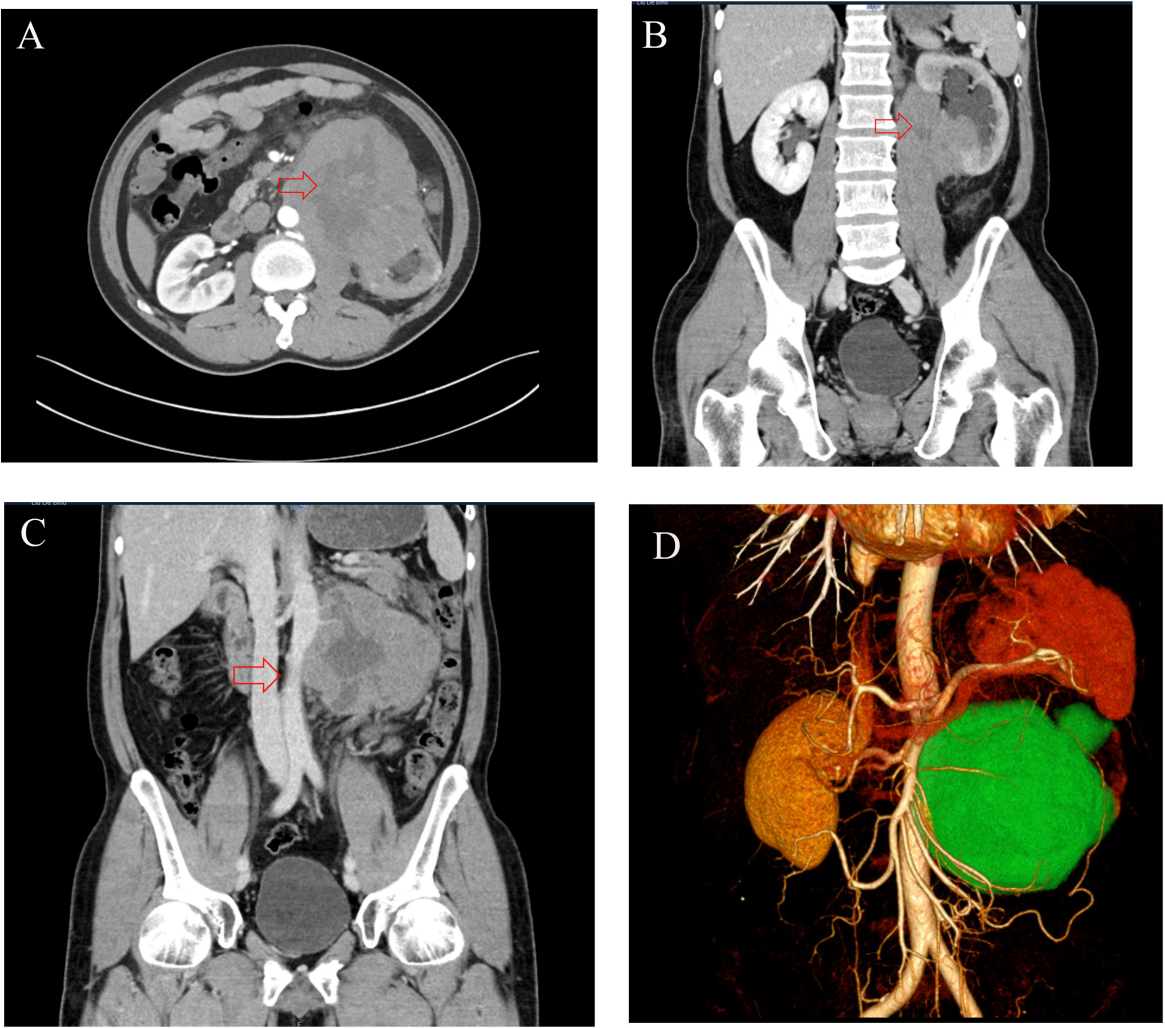
**(A, B)** The CT imaging revealed a large (115 × 120 × 97 mm) hypodense mass at the lower pole of the left kidney with internal heterogeneity, accompanied by left hydronephrosis and ureteral obscuration. **(C, D)** The CT imaging demonstrates a mass encircling the abdominal aorta and left renal artery/vein, with blood supply derived from branches of the left renal artery, superior mesenteric artery, and inferior mesenteric artery (in panel **D**, the tumor is labeled in green, the kidney is in brown, and arteries are in red).

The patient presented to our institution on 24 February 2024 with a chief complaint of persistent left flank pain lasting more than 1 month. Physical examination upon admission revealed an empty left scrotal sac and paroxysmal pain in the left lumbar region that worsened in the left lateral decubitus position with radiation to the left shoulder. Contrast-enhanced abdominal CT with three-dimensional reconstruction (24 February 2024) revealed a large mass (115 × 120 × 97 mm) in the inferior pole of the left kidney, with obscured visualization of the left ureter and associated hydronephrosis suggestive of renal malignancy. Multiple enlarged lymph nodes (maximum diameter approximately 10 mm) were observed in the peri-tumoral peritoneal and retroperitoneal regions (**Figures 1A, B**). Three-dimensional computed tomographic angiography of the upper abdominal great vessels (28 February 2024) revealed encasement of the abdominal aorta and left renal artery and vein by the left renal tumor, with tumor vascular supply originating from the left renal artery along with branching vessels from both the superior and inferior mesenteric arteries (**Figures 1C, D**).

**Figure 2:**
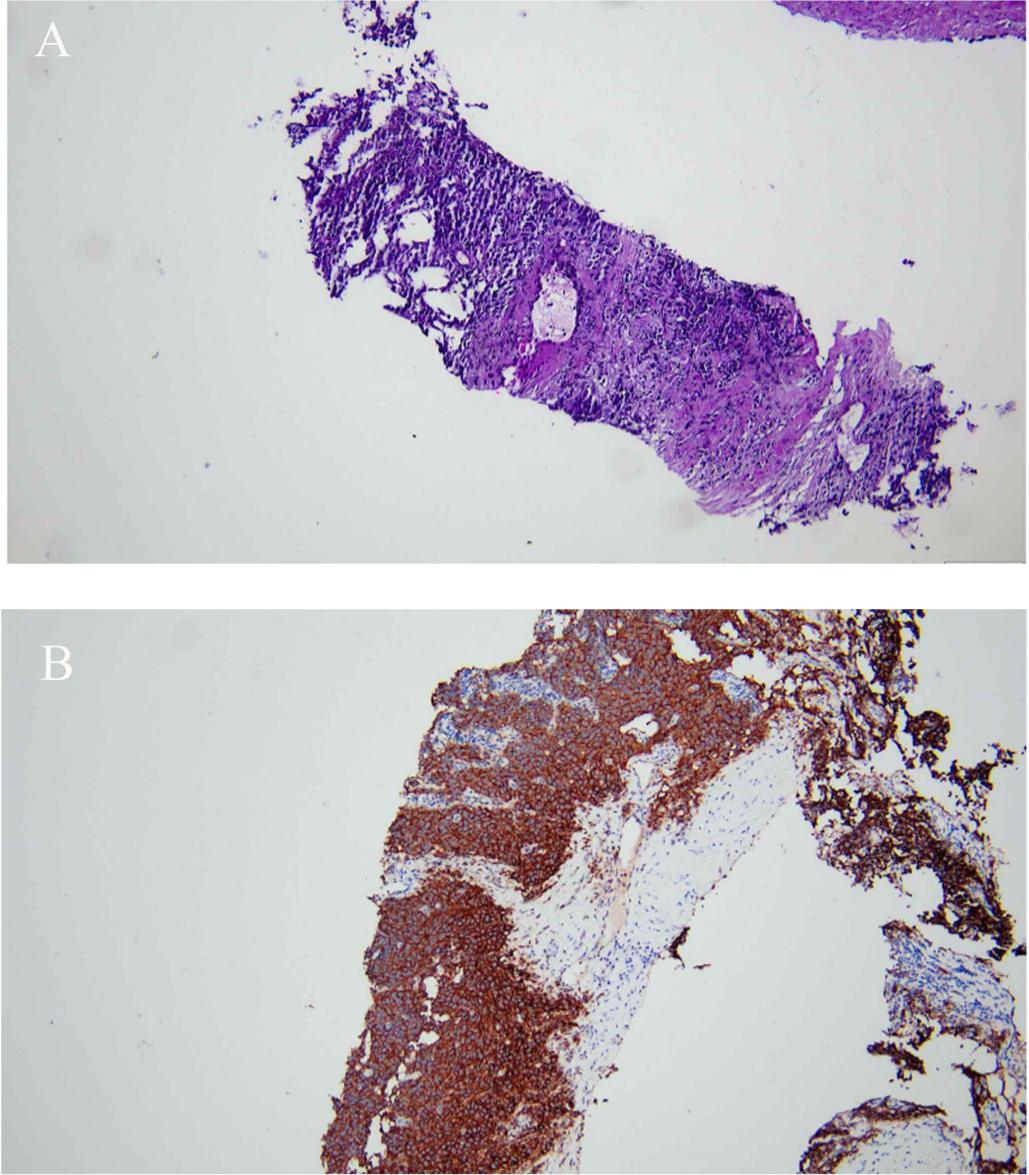
**(A, B)** H&E, ×40. Histopathological examination of the left renal biopsy revealed an infiltration of atypical cells with necrotic foci in fibrous stroma, supported by positive PLAP staining, consistent with a diagnosis of seminoma.

On 29 February 2024, a percutaneous core needle biopsy of the left renal mass was performed under local anesthesia. Histopathological examination confirmed metastatic seminoma, consistent with testicular primary origin (**Figure 2A**).

Immunohistochemical analysis showed D2-40 (+), 0CT3/4 (+), PLAP (+), SALL4 (+), CD10 (+), TdT (+), CD20 (B cell +), CD79a (B cell +), Pax-5 (B cell +), CD3 (T cell +), CD5 (T cell +), CD3ϵ (T cell +), CD43 (T cell+), and Ki67 (~60%+) (**Figure 2B**). The patient was advised to undergo chemotherapy initially, with surgical intervention to be considered after tumor shrinkage. Postoperative follow-up (13 March 2024) showed AFP = 4.8 ng/mL (normal reference interval, ≤7.0 ng/mL), human chorionic gonadotropin (hCG-β) = 186 IU/L (normal reference interval, 0–5 IU/L), and lactate dehydrogenase (LDH) = 375 U/L (normal reference interval, 120–250 U/L).

**Figure 3:**
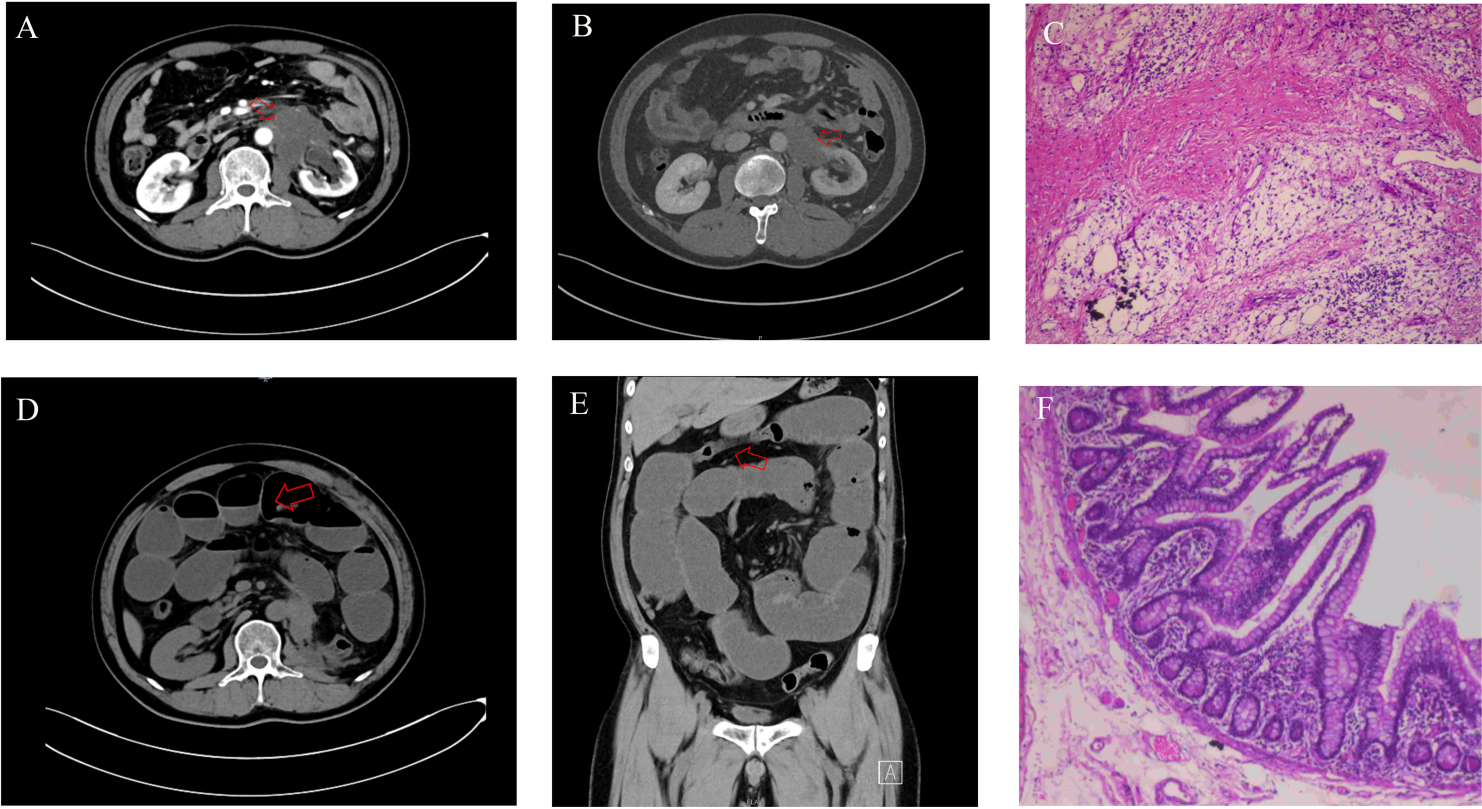
**(A)** The CT imaging of the whole abdomen before the third BEP chemotherapy showed that the 58 × 62 × 70 mm mass at the lower pole of the left kidney was smaller than before. **(B)** Preoperative abdominal CT imaging revealed a 56 × 45 × 60 mm mass at the lower pole of the left kidney, demonstrating significant size reduction compared to pre-chemotherapy scans. **(C)** H&E, ×40. Histological examination after left nephrectomy showed inflammatory cell infiltration, a few areas of lymphoid tissue hyperplasia, lymphoid follicle formation, and no tumor cells. **(D, E)** Abdominal CT demonstrated small bowel dilatation with air-fluid levels, indicative of small bowel obstruction. **(F)** H&E, ×100. Histopathological examination of the resected obstructed bowel segment revealed no evidence of tumor cells. BEP, bleomycin, etoposide, and cisplatin.

The patient began treatment with the BEP chemotherapy regimen on 29 March 2024 and completed four cycles. During chemotherapy, CT scans of the chest, abdomen, and pelvic organs were performed, revealing no evidence of new recurrent tumor lesions. On 12 May 2024, follow-up testing demonstrated serum tumor marker levels of AFP = 6.0 ng/mL, hCG-β < 1.2 U/L, and LDH = 177 U/L. A contrast-enhanced three-dimensional abdominal CT performed on 15 May 2024 demonstrated a reduction in the left renal mass to 58 × 62 × 70 mm, with multiple enlarged lymph nodes remaining in the peritoneal and retroperitoneal regions (**Figure 3A**). The contrast-enhanced abdominal CT with three-dimensional reconstruction performed on 17 October 2024 revealed a reduction in the left renal mass to 56 × 45 × 60 mm, along with a decreased size of the abdominal and retroperitoneal lymph nodes compared to previous imaging (**Figure 3B**).

AFP = 6.3 ng/mL, hCG-β < 1.2 U/L, and LDH = 1,76 U/L were examined on 15 October 2024. However, chemotherapy failed to eradicate the metastatic tumor tissue. To prevent tumor recurrence and metastasis, as well as to prolong the patient’s survival and improve quality of life, a left nephrectomy with partial resection of retroperitoneal enlarged lymph nodes was performed on 1 November 2024, following a comprehensive evaluation. Postoperative histopathological analysis revealed fibroplasia in the left renal tissue and adjacent perirenal adipose tissue, accompanied by prominent chronic inflammatory cell infiltration. Focal lymphoid hyperplasia with follicular formation was observed, consistent with chemotherapy-induced changes. Repeated sampling and thorough examination confirmed no definitive evidence of residual tumor cells (**Figure 3C**).

Twelve days after the left nephrectomy, the patient developed abdominal distension accompanied by cessation of flatus and defecation. On 13 November 2024, the patient was admitted to our institution for evaluation. Contrast-enhanced abdominal CT with three-dimensional reconstruction revealed small bowel dilation with gas-fluid levels, consistent with a diagnosis of small bowel obstruction (**Figures 3D, E**). The attending physician initially diagnosed acute complete intestinal obstruction secondary to either peritoneal tumor metastasis or post-nephrectomy adhesions, warranting emergent surgical exploration and intervention. On 14 November 2024, the patient ultimately underwent exploratory laparotomy with resection and anastomosis of the obstructed small bowel. Pathological examination of the resected specimens demonstrated no evidence of tumor cells (**Figure 3F**). The patient resumed normal anal flatus and bowel movements and was discharged 10 days postoperatively.

**Figure 4:**
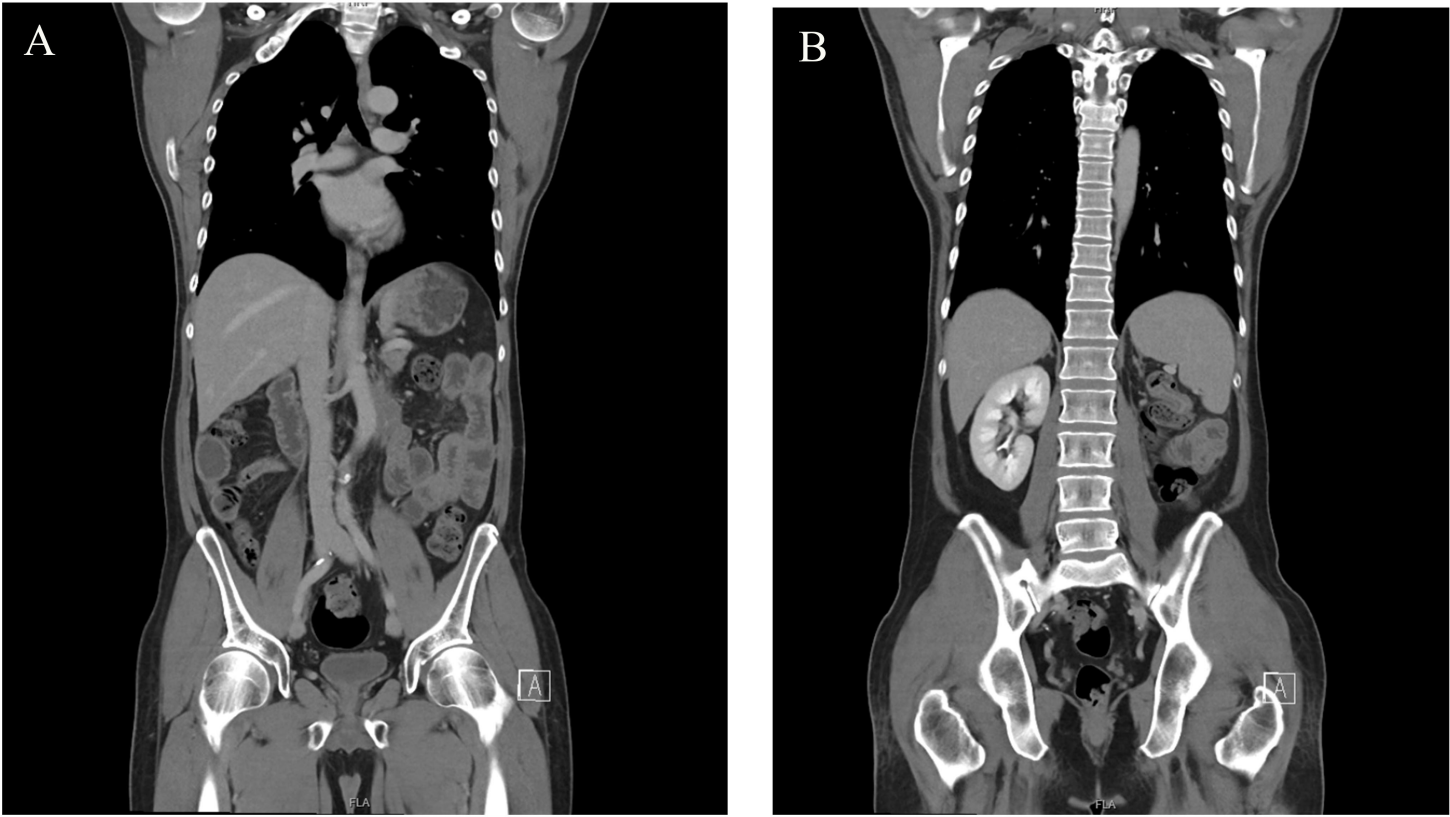
**(A, B)** Four months post-nephrectomy, contrast-enhanced abdominal CT with three-dimensional reconstruction demonstrated no abnormal lymphadenopathy or masses in the abdominal cavity.

During the follow-up visit on 6 March 2025, a contrast-enhanced abdominal CT with three-dimensional reconstruction revealed no abnormalities in the abdominal cavity or organs (**Figures 4A, B**). On 7 March 2025, AFP = 6.2 ng/mL, hCG-β < 1.2 U/L, LDH = 138 U/L. The patient has achieved an excellent recovery with no evidence of tumor recurrence on the current evaluation.

## 3 Discussion

Testicular germ cell tumors (TGCTs) are malignant tumors commonly seen in male individuals aged 14–44 years, including germ cell neoplasia *in situ* (GCNIS) and non-GCNIS, with GCNIS including seminoma and non-seminoma (14, 15). The incidence of TGCT has continued to increase globally over the past 20 years, with cryptorchidism leading to a three- to sixfold increased risk of developing TGCT (14, 16). The case reported in this article has a cryptorchidism, which not only is a high-risk factor for testicular tumors but also adds to the complexity of diagnosis and therapy. Mamsen LS et al. (17) demonstrated that early orchidopexy (performed at 0.3 to 3.8 years of age) significantly reduces the risk of subsequent infertility in treated patients. Ergül RB et al. (18) conducted a retrospective study of 23 post-pubertal patients undergoing orchiectomy for cryptorchidism, identifying one seminoma case while demonstrating absent normal spermatogenesis in all undescended testes. For post-pubertal male individuals with unilateral cryptorchidism, early orchiectomy should be considered, with semen cryopreservation from the contralateral testis recommended for those desiring future fertility (11). However, this patient from an impoverished region experienced a delayed diagnosis of left cryptorchidism due to limited health awareness, family poverty, and inadequate medical resources. This case demonstrates the challenges of cryptorchidism management and TGCT prevention in resource-limited settings. Although sperm cryopreservation could preserve fertility, the lack of relevant support prevented its implementation, highlighting the urgent need for enhanced medical education and services in low-income regions.

### 3.1 Symptoms and diagnosis of testicular seminoma

The majority of patients with testicular seminoma manifest a painless testicular mass (8). Additionally, a rare proportion of patients are also detected with atypical presentations such as abdominal or inguinal masses (19, 20), inguinal hernia (21, 22), and testicular torsion (23). In cytologic features, seminoma morphologically resembles the large cells of GCNIS, with slight pleomorphism, clear cytoplasm and deep staining, and eccentric nuclei with one or more prominent nucleoli, and the cells are arranged in small nests or sheets (8, 14). Immunohistochemical features were predominantly CD117 (+), whole cell keratin (+), NANOG (+), OCT3/4 (+), PLAP (+), podoplanin (+), PRAM (+), and SOX17 (+) (8). This case presented with a painful left inguinal mass and was pathologically confirmed as seminoma postoperatively. For similar cases, we recommend an initial assessment for scrotal emptiness with imaging studies to differentiate it from an inguinal hernia. If testicular tissue is obtained, a pathological examination with immunohistochemical staining should be performed to prevent diagnostic delay or misdiagnosis (12).

### 3.2 Therapy and prognosis of testicular seminoma

The standard treatment option for testicular seminoma is orchiectomy with postoperative monitoring or adjuvant chemotherapy, of which the BEP chemotherapy regimen is one of the standard regimens for the treatment of seminoma (11). Approximately 80% of patients with testicular seminoma are in clinical stage I at the time of diagnosis and are cured by orchiectomy and postoperative chemotherapy (12). A retrospective study analyzing 1,344 patients with clinical stage I testicular seminoma after therapy found a recurrence rate of approximately 13%, a median time to recurrence of 14 months, and recurrence in approximately 92% of patients concentrated within 3 years of diagnosis (24). In documented clinical cases, carboplatin has served as an effective treatment for stage I seminoma, demonstrating reduced nephrotoxicity and ototoxicity compared to cisplatin while necessitating vigilance against acute kidney injury (10). Additionally, adjuvant chemotherapy is the primary treatment modality for most patients with clinical stage II seminoma (25). The patient in this case was diagnosed with clinical stage I testicular seminoma but did not undergo postoperative chemotherapy due to multiple socioeconomic factors, resulting in the rapid development of renal metastasis. Based on the evidence, it can be hypothesized that the patient’s failure to receive adjuvant chemotherapy postoperatively was a significant contributing factor to the rapid development of tumor distant metastasis.

### 3.3 Renal metastasis from seminoma of the testis

Testicular seminoma typically metastasizes via lymphatic pathways to the abdominal and retroperitoneal lymph nodes, while renal metastasis occurs in less than 1% of cases (26, 27). In the series of 650 patients with testicular tumors reported by Husband JE et al. (28), renal metastasis was observed in only six cases (0.9%). Naimi A et al. (29) reported a rare case of testicular seminoma metastasizing to both the kidney and cervical lymph nodes 25 years after initial orchiectomy and adjuvant chemotherapy, with the patient succumbing within 1 month of metastasis diagnosis. For patients with metastatic seminoma presenting with limited retroperitoneal lymph node involvement, retroperitoneal lymph node dissection (RPLND) demonstrates excellent short-term outcomes, with 24-month progression-free survival (PFS) and overall survival (OS) rates reaching 90% and 100%, respectively (30). The European Association of Urology’s latest guidelines recommend RPLND for patients with clinical IIA/B seminoma (31). The International Germ Cell Cancer Collaborative Group (IGCCCG) recommends that intermediate- and low-risk patients with distant metastases are required to receive four cycles of BEP chemotherapy, and high-risk patients may be treated with three cycles of BEP regimen (11, 12). This case involved a patient with testicular seminoma who developed early recurrence manifested by left renal and retroperitoneal lymphadenopathy metastasis. Following four cycles of BEP chemotherapy, significant tumor regression was achieved in both renal lesion and nodal metastases, ultimately permitting successful surgical intervention with left radical nephrectomy and RPLND. Beyer J et al. (32) conducted a multicenter analysis of 2,402 metastatic seminoma patients treated with either BEP or EP regimens. The study demonstrated significant improvements in PFS and OS following chemotherapy. Notably, pretreatment LDH levels exceeding 2.5 times the upper limit of normal (ULN) were identified as an independent poor prognostic factor in this metastatic cohort. The American Society of Clinical Oncology (ASCO) identifies three key serum markers for seminoma: elevated AFP, increased β-hCG, and LDH exceeding 1.5 times normal levels. These markers typically indicate greater tumor aggressiveness and metastatic potential, strongly correlating with worse outcomes (33). Follow-up at 4 months post-treatment revealed normal serum levels of AFP, β-hCG, and LDH in this patient, suggesting a favorable prognosis with likely prolonged PFS and OS.

### 3.4 Literature summary and case uniqueness

Testicular seminoma with renal metastasis is exceptionally rare, with only sporadic cases reported in the literature. **Table 2** summarizes recently reported cases of testicular seminoma with renal metastases, including patient age, clinical history/symptoms, treatment regimens, and outcomes. Compared with previously reported cases in the literature, the distinctive features of this case lie in the patient’s history of cryptorchidism, rapid recurrence due to omission of adjuvant chemotherapy postoperatively, and remarkable therapeutic response to BEP chemotherapy. The patient in the case was found to have metastatic seminoma through an ultrasound-guided percutaneous biopsy of the left renal mass. Immunohistochemical analysis revealed positive expression of CD117, OCT3/4, Ki-67, NANOG, PLAP, and PRAM, while being negative for AFP and β-hCG, providing essential evidence for differential diagnosis.

**Table 2:** Summary of reported cases of renal metastasis from testicular seminoma.

Patient age	Clinical history/symptoms	Treatment regimen	Pathological diagnosis/tumor markers	Treatment outcomes	Contributing authors
36 years	The patient presented with right testicular enlargement. CT revealed enlarged para-aortic and paracaval lymph nodes with right renal and adrenal masses	The patient underwent unilateral orchiectomy combined with right nephrectomy/adrenalectomy and retroperitoneal lymph node dissection, followed by four cycles of BEP chemotherapy	Testicular seminoma (Stage IIIB, pT2pN2M0 S2) with concurrent clear cell renal cell carcinoma (pT2a R0 G2), pathologically confirmed nodal metastasis, normal β-hCG/AFP with elevated LDH	Three years after chemotherapy, the patient developed left renal and pelvic bone metastases from clear cell renal cell carcinoma of the right kidney	Auskalnis S et al. (34)
46 years	The left renal mass was detected 7 months ago.	BEP chemotherapy	Stage IIC seminoma. Vimentin (partial+), CD117 (+), EMA (focal weak+), CD34 (endothelial+), S-100 (−), Desmin (−), LCA (−), GATA-3 (−), Ki-67 (60%)	The patient achieved significant tumor reduction following chemotherapy	Song J et al. (27)
48 years	Left testicular seminoma metastasized to the kidney and cervical lymph nodes after a 25-year disease-free interval	The patient had a history of left orchiectomy and adjuvant chemotherapy 25 years prior. Upon detection of metastasis, surgical intervention and chemotherapy were administered	Metastatic seminoma. Immunohistochemical staining was positive for PLAP, CD117, CD10, Ki-67, and OCT3/4	The patient succumbed to the disease 1 month after the diagnosis of metastatic seminoma	Naimi A et al. (29)
34 years	The patient underwent CT scanning for chronic leg and back pain, which revealed a right renal mass	The patient underwent laparoscopic radical nephrectomy. Scrotal ultrasound and subsequent right orchiectomy also confirmed the presence of seminoma	Seminoma	Not mentioned	Hadley DA et al. (35)
28 years	The patient presented with a 1-year history of painless left testicular mass. Contrast-enhanced CT revealed a tumor thrombus in the left renal vein	The left testis was resected via an inguinal approach, followed by four cycles of salvage chemotherapy postoperatively	Seminoma (Stage pT2)	No evidence of recurrence was found during the 11-month postoperative follow-up	Effert PJ et al. (36)

BEP, bleomycin, etoposide, and cisplatin; AFP, alpha-fetoprotein; hCG-β, human chorionic gonadotropin; LDH, lactate dehydrogenase.

### 3.5 Limitations

This study represents a single-case report, so the generalizability and reliability of its findings are inherently limited. Furthermore, as this study was conducted retrospectively, it lacks certain detailed imaging data, histopathological images, and gross specimen photographs, which may limit the comprehensive analysis of the case. Future studies should incorporate larger patient cohorts and integrate multicenter data to validate the findings of this research.

## 4 Conclusions

Renal metastasis from recurrent testicular seminoma is exceptionally rare, making early diagnosis and standardized treatment critical for optimizing patient outcomes. In medically underserved and economically disadvantaged regions, timely management of cryptorchidism and targeted patient education are fundamental for TGCT prevention. For patients with metastatic seminoma, combined BEP chemotherapy and surgical intervention significantly improve clinical outcomes.

## Data Availability

The original contributions presented in the study are included in the article/**Supplementary Material**. Further inquiries can be directed to the corresponding author.
